# Influence of maternal psychological distress during COVID-19 pandemic on placental morphometry and texture

**DOI:** 10.1038/s41598-023-33343-4

**Published:** 2023-05-10

**Authors:** Haleema Saeed, Yuan-Chiao Lu, Nickie Andescavage, Kushal Kapse, Nicole R. Andersen, Catherine Lopez, Jessica Quistorff, Scott Barnett, Diedtra Henderson, Dorothy Bulas, Catherine Limperopoulos

**Affiliations:** 1 Department of Obstetrics & Gynecology, MedStar Washington Hospital Center, Washington, DC 20010 USA; 2 Developing Brain Institute, Children’s National Hospital, 111 Michigan Ave. NW, Washington, DC 20010 USA; 3 Division of Neonatology, Children’s National Hospital, Washington, DC 20010 USA; 4 Present Address: Division of Radiology, Children’s National Hospital, Washington, DC, 20010 USA

**Keywords:** Magnetic resonance imaging, Paediatrics, Human behaviour

## Abstract

The Coronavirus Disease 2019 (COVID-19) pandemic has been accompanied by increased prenatal maternal distress (PMD). PMD is associated with adverse pregnancy outcomes which may be mediated by the placenta. However, the potential impact of the pandemic on in vivo placental development remains unknown. To examine the impact of the pandemic and PMD on in vivo structural placental development using advanced magnetic resonance imaging (MRI), acquired anatomic images of the placenta from 63 pregnant women without known COVID-19 exposure during the pandemic and 165 pre-pandemic controls. Measures of placental morphometry and texture were extracted. PMD was determined from validated questionnaires. Generalized estimating equations were utilized to compare differences in PMD placental features between COVID-era and pre-pandemic cohorts. Maternal stress and depression scores were significantly higher in the pandemic cohort. Placental volume, thickness, gray level kurtosis, skewness and run length non-uniformity were increased in the pandemic cohort, while placental elongation, mean gray level and long run emphasis were decreased. PMD was a mediator of the association between pandemic status and placental features. Altered in vivo placental structure during the pandemic suggests an underappreciated link between disturbances in maternal environment and perturbed placental development. The long-term impact on offspring is currently under investigation.

## Introduction

Coronavirus disease 2019 (COVID-19) is a novel infectious disease first reported in a few cases of pneumonia in Wuhan, China on Dec. 31, 2019^1^. Caused by the severe acute respiratory syndrome coronavirus 2 (SARS-CoV-2), infections spread globally, and COVID-19 was characterized by the World Health Organization as a pandemic on March 11, 2020^2^. In addition to concerns about acquiring COVID-19, pregnant women are exposed to pandemic related stressors, including social distancing, generalized anxiety, financial insecurity, and fear of mortality^3,4^. The impact of these exposures on intrauterine development remains unknown. The COVID-19 pandemic offers a novel window for a natural history investigation of profound lifestyle changes and the effects of maternal stress on placental development.

Maternal mental health concerns are considered a major public health challenge^5^. Epidemiological studies suggest that even pre-pandemic severe maternal stress is associated with poor pregnancy and neonatal outcomes^6–8^. Periods of hardship, such as natural disasters, famine or military invasion, can cause low birth weight, preterm delivery, perinatal anxiety or depressive symptoms as well as cognitive and behavioral problems later in life^9–14^. A proposed mechanism underlying these adverse outcomes is placental dysfunction as a result of maternal stress^9,15^. The placenta is the primary interface between mother and fetus, and plays important role in nutrition, excretion of toxins, immunity, and metabolism^16^. It is susceptible to a hostile uterine environment leading to altered placenta function and development^17,18^.

At this time, there are limited non-invasive tools to assess placental development in utero, especially prior to the onset of fetal compromise. Detailed magnetic resonance imaging (MRI) analyses of placentas, a recent innovation, can identify global and local differences in placental structure and microstructure^19–21^. Specifically, the morphometric and textural analyses of placental development have been utilized to study pregnancy complications^21–24^. For example, pregnancies complicated by fetal growth restriction showed smaller placental volumes and increased heterogeneity of the placenta compared to healthy controls^24^. Textural analyses in biomedical imaging has been used to quantify tissue microstructure in healthy and diseased tissues^25–28^ and is a proposed method to identify regional changes related to injury or inflammation^29^. The placental textural features calculated from in vivo placental MR imaging represent a new era of advanced placental tissue characterization^30^. This suggests a potential role for antenatal MR imaging to provide novel insights to placental development in high-risk conditions.

The aim of this study was to examine the impact of the COVID-19 pandemic on in vivo structural and textural placental development using MRI. We hypothesize that increased maternal mental distress^31^ in pregnant women during the COVID-19 pandemic would lead to changes in placental structure.

## Methods

### Recruitment

Women with singleton pregnancies were recruited in an ongoing prospective observational study between June 2020 to April 2021, as part of Project RESCUE (Reducing Elevated Stress from COVID-19 Exposure) at Children’s National Hospital. The inclusion criteria included women older than 17 years with singleton pregnancies of greater than 8 weeks gestational age (GA). Exclusion criteria included women with pregnancies complicated by known chromosomal syndromic conditions, inability to enter the MRI scanner due to physical or psychological reasons, or high-risk pregnancy conditions such as diabetes and hypertensive disorders.

This study was approved by the Children’s National Hospital Institutional Review Board (IRB). All methods were performed accordance with the relevant guidelines and regulations. Pre-pandemic pregnant women were enrolled between March 2014 and February 2020 as part of an observational cohort study examining normal brain development in low-risk pregnancies, also approved by Children’s National Hospital IRB. Recruitment methods and eligibility criteria were identical for both cohorts. Written, informed consent was obtained from each participant for both cohorts.

### Clinical data and maternal stress questionnaires

Clinical and demographic data were collected and/or abstracted from the medical record for each participant, including maternal age, maternal weight at MRI, parity, GA at birth, birth weight, and race/ethnicity.

All pregnant women were administered four questionnaires on the day of the MRI visit, including Perceived Stress Scale (PSS), Edinburgh Postnatal Depression Scale (EPDS), Spielberger State Anxiety Inventory (SSAI), and Spielberger Trait Anxiety Inventory (STAI). The PSS is a widely used measurement of the degree of stressful feelings experienced in the past month^32^. The questionnaire comprises 10 questions, with scoring ranging from 0 to 40. A score greater than 15 indicates a higher-than-average level of stress^32^. The EPDS (range: 0 to 30) is a useful tool to identify patients at risk for developing postpartum depression^33^. A score greater than 10 indicates a higher risk fordepression^34^. The SSAI was designed to evaluate anxiety as emotional state (range: 20 to 80) and as a personality trait (STAI, range: 20 to 80)^35^. A score higher than 40 indicates the presence of anxiety^36,37^.

### MRI data acquisitions

Axial single shot fast spin echo T2-weighted images were acquired on a 1.5 Tesla Discovery MR450 scanner (GE Healthcare, Milwaukee, WI) using an eight-channel surface receiver coil. Acquisition parameters were as follows: echo time = 160 ms, repetition time = 1100 ms, field of view = 420 × 420 mm, and slice thickness = 4 mm. The final in-plane resolution was 1.64 × 1.64 mm with 40–60 consecutive slices for full placental coverage. Neither sedation nor contrast was used during MRI studies. Each subject was scanned up to two time points in the fetal period.

### MRI data post-processing

**Figure 1:**
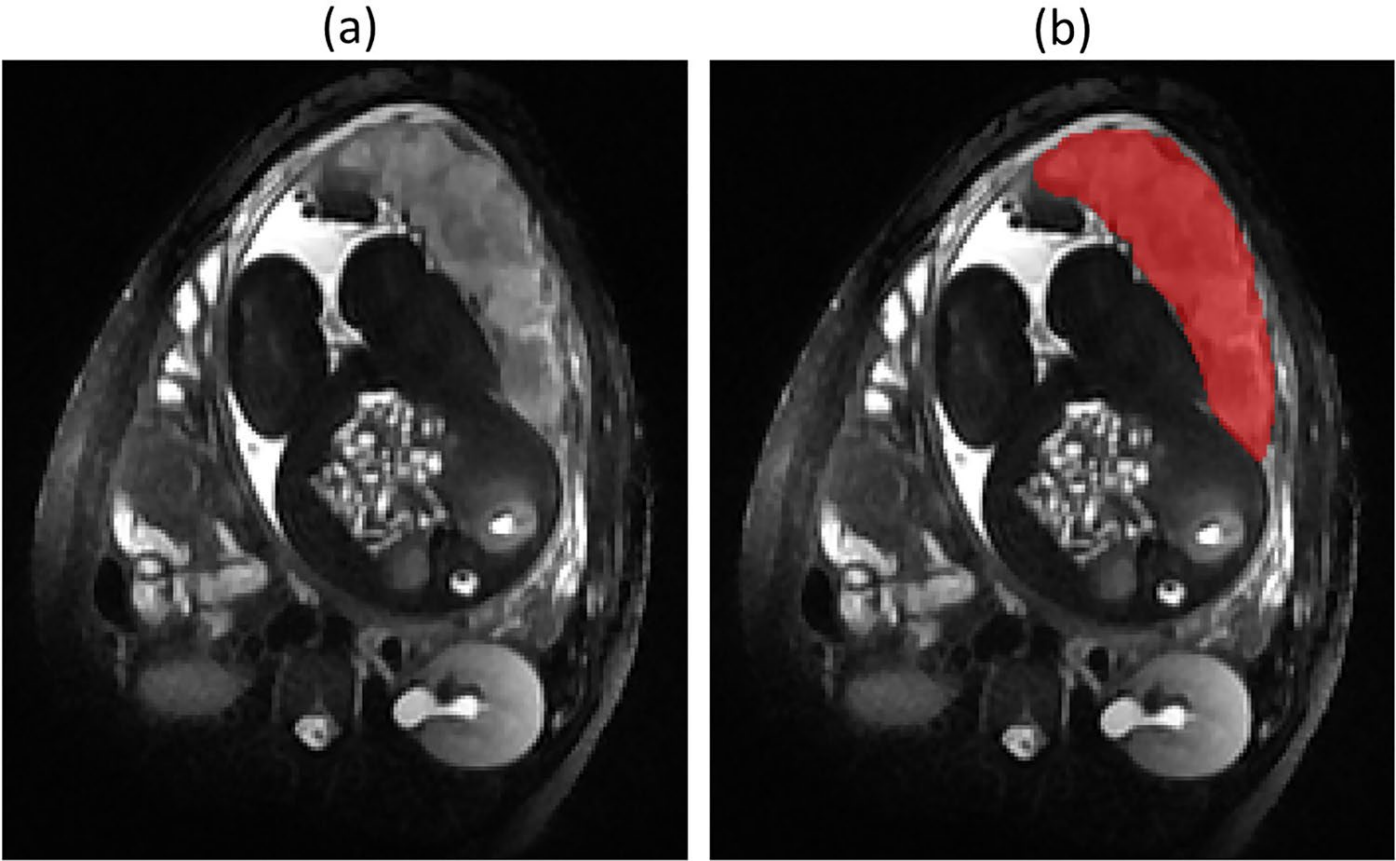
Image segmentation of a representative placenta. (**a**) T2-weighted placenta image; (**b**) Manual segmentation of the placenta.

Placentas were manually segmented in the plane of acquisition and then corrected on the other planes to ensure spatial consistency using ITK-SNAP (Fig. 1)^38^. The segmentations were performed by two research engineers (K.K., N.R.A.) with 3–5 years of experience in MRI placental segmentation and were reviewed by a senior neonatologist (N.A.) with more than 7 years’ experience. Intra-rater and inter-rater reliability of the placenta segmentations were determined using 30% of randomly selected scans (80 pre-pandemic and 27 pandemic) by the two trained raters (K.K., N.R.A.). Intra- and inter-rater reliabilities using intra-class correlation coefficient were all higher than 0.97 for both pre-pandemic and pandemic cohorts. All were blinded to pandemic vs. pre-pandemic cohort.

### Shape features

To characterize three-dimensional (3-D) placental shape, three shape features were used: volume, thickness, and elongation. Detailed elaborations of the determination of these three features have been provided in Dahdouh and Andescavage’s studies^22,24^, and brief descriptions of the features are summarized here. The volume was calculated based on the triangular meshes of the 3-D placental model^22^. Thickness was defined as the maximal distance between the points of the placenta belonging to the maternal surface and their projection on the fetal surface^22^. Elongation was defined as the length of the longest branch of the 3-D medial axis skeleton of the shape^22^. All calculations were implemented in MATLAB R2019a (The MathWorks, Inc., Natick, MA).

### Textural features

To characterize the placental gray level (GL) appearance, three sets of textural features were used. We followed the analytical pipeline proposed by Dahdouh et al. to identify these three sets of placental textural features^22^. Detailed descriptions can be found in Dahdouh’s study, and here we briefly describe these features^22^. The first set of placental textural features included the mean, variance, kurtosis, and skewness of the GL distribution of the placenta^22^. These four measures were designed to determine the variation of GL intensity of the placenta and were normalized by their corresponding values on the whole image. The second set of placental textural features included energy, entropy, inverse difference moment, contrast, cluster shade and cluster prominence, which were calculated based on the GL Co-occurrence Matrix (GLCM)^39,40^. These six features were characterized by capturing the spatial dependencies between pairs of GL, and each feature was averaged over all directions. The third set of placental textural features included short run emphasis, long run emphasis, GL non-uniformity, run length non-uniformity, low GL run emphasis, high GL run emphasis, short run low GL emphasis, short run high GL emphasis, long run low GL emphasis and long run high GL emphasis. These 10 features were computed based on the run-length statistics^41,42^. The run-length matrix counted the number of “runs” of consecutive voxels in a given direction^41^. An image with a high number of short runs is interpreted as finer texture with more details than an image with high number of long runs^41^. All textural feature calculations were implemented in MATLAB R2019a (The MathWorks, Inc., Natick, MA).

### Statistical analyses

Univariate analyses were performed to explore the demographic data. The Kolmogorov–Smirnov test was first utilized to test the normality of the continuous variables, including GA; maternal age; maternal weight at MRI; and birthweight, and the results showed that all these variables were not normally distributed. The fetal and maternal demographics were therefore compared between pre-pandemic and pandemic cohorts using Wilcoxon–Mann–Whitney tests for GA, maternal age, maternal weight at MRI and birthweight and using Chi-square tests for fetal sex, number of scans, maternal parity, maternal race/ethnicity and the temporal distribution of subject recruitment.

The generalized estimating equation (GEE) was utilized to determine the following associations for comparing the pre-pandemic and pandemic cohorts^43^. First, the associations between pandemic status (pre-pandemic: 0; pandemic: 1) and maternal distress measures (SSAI, STAI, PSS and EPDS) were determined using GEE, adjusting for GA at MRI (weeks), given the known effects of GA on placental MRI features. Second, the associations between pandemic status and placental shape features were analyzed using GEE. The primary independent variable was the pandemic status (pre-pandemic: 0; pandemic: 1), and all GEEs were adjusted for GA at scan (weeks). Third, the associations between pandemic status and placental textural features were analyzed using GEE, adjusting for GA at scan (weeks). In addition, we further adjusted maternal distress (low distress: 0; high distress: 1) in the GEEs to determine whether maternal distress is a mediator in the association between pandemic status and placental shape and textural features. High distress was defined as any one of the four distress measure summary scores being greater than their corresponding threshold (SSAI: 40; STAI: 40; PSS: 15; EPDS: 10)^32,34,36,37^. Furthermore, the association between placental features and birth weight (g) by pandemic status were also investigated using GEEs, adjusting for GA at MRI (weeks), maternal distress (0: low distress, 1: high distress,) and GA at birth (weeks). Mediation analyses were further implemented to determine whether prenatal maternal distress works as a mediator on pandemic status and placental features^44^. Three steps of mediation analyses were conducted using GEE: (1) the association between placental features and the pandemic status, adjusting for GA at MRI; (2) the association between maternal distress measures and the pandemic status, adjusting for GA at MRI; (3) the association between placental features and the pandemic status, adjusting for GA at MRI and the significant maternal distress measures found in the previous step. Sensitivity analyses were conducted on two additional covariates, fetal sex and maternal weight, and their effect on the placental features were examined. Lastly, the time trend was investigated by fitting two GEE models (one before the pandemic and the other during the pandemic) to explore the associations between placental features and date of evaluation, adjusting for gestational age at MRI (weeks). The time trend throughout the study period was fitted using nonlinear mixed-effects estimation with the quadratic spline function^45^. The critical value for statistical significance was set as 0.05. The q-values calculated by the false discovery rate method for the number of features in each set (3 for shape features; 4 for the first set, 6 for the second set and 10 for the third set of textural features) were also reported to reflect significant parameters under multiple comparisons^46^. The unstructured correlation matrix was utilized in all GEE models, with the robust sandwich covariance matrix^47^. All analyses performed in this study were conducted using MATLAB R2019a (The MathWorks, Inc., Natick, MA, USA), and all hypothesis tests were 2-sided.

## Results

### Demographics

**Figure 2:**
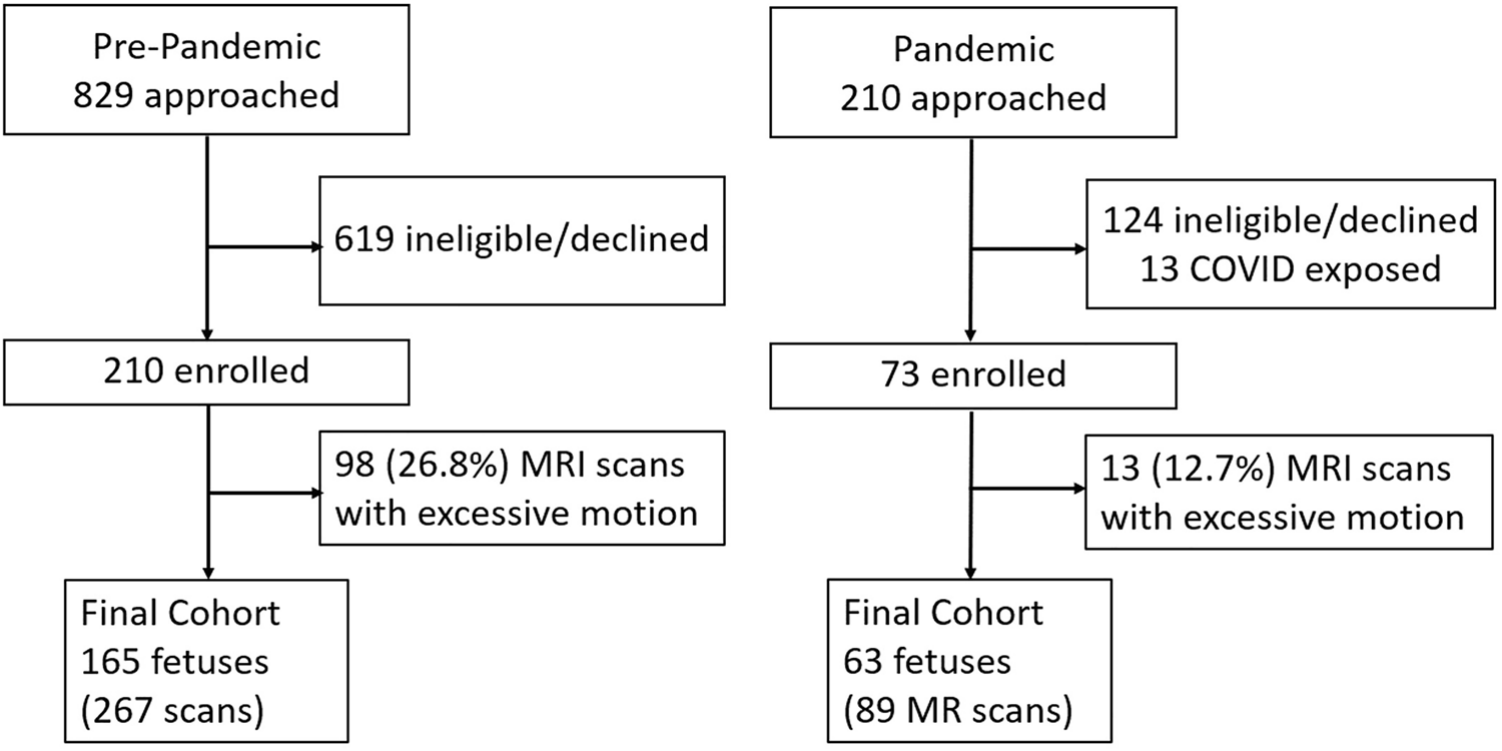
Flow diagram summarizing our subject recruitment in this study. Eligible women were recruited from community maternal fetal medicine offices and referred by their obstetrics providers. The pre-pandemic cohort was recruited between March 2014 and February 2020, and the pandemic cohort was recruited between June 2020 to April 2021. The study team spoke to potential participants alongside the obstetricians and would follow up with those interested in participation. Written informed consent was obtained from all participants before completing study procedures.

Participant recruitment is shown in Fig. 2. One hundred and eleven (23.8%) MRI scans with excessive motion were excluded. The final data set consisted of 228 pregnant women between 16.7 to 39.4 gestational weeks, in which a total of 356 placenta MRI scans were acquired (Table 1). Among the 228 study participants, 128 were scanned at two time points during pregnancy (102 pre-pandemic and 26 pandemic) while all other subjects were scanned once (63 pre-pandemic and 37 pandemic). The temporal distribution of subjects recruited is shown in the Supplementary Fig. 5. The median GA at MRI was 29.3 weeks (range: 16.7 to 39.4) for the pre-pandemic cohort and was 30.1 weeks (range: 17.0 to 38.4) for the pandemic cohort. The median maternal age was 34.4 years old (range: 17.0 to 50.7). The median GA at birth was 39.6 weeks (range: 31.0 to 41.9), and the median birth weight was 3.35 kg (range: 1.02 to 4.87). The maternal distress measures, PSS and EPDS, were higher in the pandemic cohort (Table 2).

**Table 1:** Demographics of 228 pregnant women who underwent 356 prenatal MRI studies.

N [%] or median [IQR]	All subjects	Pre-pandemic	Pandemic	p
Number of subjects	228	165	63	
Female fetus	103 [48]	71 [46]	32 [54]	0.29
Male fetus	110 [52]	83 [54]	27 [46]	
With 1 scan	100 [44]	63 [38]	37 [59]	** < 0.01**
With 2 scans	128 [56]	102 [62]	26 [41]	
Number of MR scans	356	267	89	
Time point 1	206 [58]	162 [61]	44 [49]	0.06
Time point 2	150 [42]	105 [39]	45 [51]	
GA at MRI	29.4 [26.3, 35.0]	29.3 [26.6, 35.3]	30.1 [25.3, 33.9]	0.10
Maternal age (years)	34.4 [31.0, 37.3]	34.3 [31.0, 38.0]	34.6 [30.5, 36.1]	0.55
Maternal weight at MRI (kg)	73.1 [66.4, 83.1]	73.3 [66.4, 83.3]	72.8 [66.5, 82.8]	0.86
Maternal parity (primiparous/multiparous)^a^	123/94	87/69	36/25	0.66
GA at birth (weeks)^b^	39.6 [38.6, 40.3]	39.6 [38.4, 40.4]	39.4 [38.9, 40.1]	0.82
Birth weight (kg)^c^	3.35 [3.02, 3.67]	3.38 [3.01, 3.67]	3.29 [3.06, 3.69]	0.98
Birth head circumference (cm)^d^	34.3 [33.5, 35.5]	34.0 [33.0, 35.5]	34.5 [33.7, 35.6]	0.42
Apgar score at 1 minute^e^	8 [8, 9]	8 [8, 9]	8 [8, 9]	0.91
Apgar score at 5 minutes^f^	9 [9, 9]	9 [9, 9]	9 [9, 9]	0.10
Delivery mode^g^				0.06
Vaginal	118 [69]	76 [64]	42 [81]	
Elective C-section	29 [17]	22 [18]	7 [13]	
Emergency C-section	24 [14]	21 [18]	3 [6]	
Race/ethnicity^h^				0.08
White	116 [57]	84 [56]	32 [59]	
Black	37 [18]	29 [19]	8 [15]	
Hispanic or Latino	25 [12]	18 [12]	7 [13]	
Asian or Pacific Islander	11 [5]	5 [3]	6 [11]	
Others	16 [8]	15 [10]	1 [2]	

GA: gestational age; IQR: interquartile range.

^a^Based on 217 (95%) subjects (pre-pandemic: 156; pandemic: 61).

^b^Based on 190 (83%) subjects (pre-pandemic: 131; pandemic: 59).

^c^Based on 182 (80%) subjects (pre-pandemic: 131; pandemic: 51).

^d^Based on 125 (55%) subjects (pre-pandemic: 78; pandemic: 47).

^e^Based on 167 (73%) subjects (pre-pandemic: 115; pandemic: 52).

^f^Based on 167 (73%) subjects (pre-pandemic: 115; pandemic: 52).

^g^Based on 171 (75%) subjects (pre-pandemic: 119; pandemic: 52).

^h^Based on 205 (90%) subjects (pre-pandemic: 151; pandemic: 54).

Bold p: p < 0.05.

**Table 2:** The results of the generalized estimating equations for the associations between maternal distress measures and pandemic status (0: pre-pandemic; 1: pandemic), adjusting for gestational age at MRI (weeks).

	Pre-pandemic		Pandemic		$\beta$	95% CI	p
	N	LS mean ± SE	N	LS mean ± SE			
SSAI	246	29.5 ± 2.3	73	30.7 ± 2.7	1.15	[− 1.48, 3.79]	0.39
STAI	246	31.0 ± 1.8	72	32.7 ± 2.2	1.76	[− 0.71, 4.22]	0.16
PSS	245	10.9 ± 1.3	74	14.1 ± 1.6	3.23	[1.40, 5.06]	** < 0.01**
EPDS	243	4.4 ± 0.9	73	6.0 ± 1.1	1.54	[0.39, 2.69]	**0.01**

N: number of scans; SSAI: Spielberger State Anxiety Inventory; STAI: Spielberger Trait Anxiety Inventory; PSS: Perceived Stress Scale; EPDS: Edinburgh Postnatal Depression Scale; LS mean: Least squares mean; SE: standard error. CI: confidence interval.

Bold p: p < 0.05.

### Placenta shape features and pandemic status

Our data showed that the pandemic cohort had higher placental volume (least squares mean: 637.9 vs. 594.0 cm^3^, p = 0.02) and placental thickness (least squares mean: 5.3 vs. 4.9 cm, p < 0.01) when adjusting for GA at MRI in the GEE models (Supplementary Fig. 1). In contrast, placental elongation was reduced in the pandemic cohort (least squares mean: 17.0 vs. 17.8 cm, p = 0.01) (Supplementary Fig. 1). After further adjusting for maternal distress (low vs. high distress), association of increased placental volume and thickness in the pandemic cohort remained unchanged (Table 3).

**Table 3:** The results of the generalized estimating equations for the associations between placental shape/textural features and pandemic status (0: pre-pandemic; 1: pandemic), adjusting for gestational age at MRI (weeks) and maternal distress (0: low distress, 1: high distress).

	Pre-pandemic (LS mean ± SE)	Pandemic (LS mean ± SE)	$\beta$	95% CI	p
Shape features					
Volume (cm^3^)	599.1 ± 43.3	643.5 ± 48.0	44.32	[3.77, 84.87]	0.03*
Thickness (cm)	4.9 ± 0.2	5.3 ± 0.3	0.38	[0.12, 0.64]	< 0.01*
Elongation (cm)	17.8 ± 0.6	17.3 ± 0.7	− 0.58	[− 1.28, 0.12]	0.10
Textural features (first set)					
Mean GL	4.72 ± 0.57	3.93 ± 0.60	− 0.80	[− 1.15, − 0.45]	< 0.01*
Variance GL	0.70 ± 0.08	0.66 ± 0.09	− 0.03	[− 0.09, 0.03]	0.28
Kurtosis GL	0.14 ± 0.03	0.23 ± 0.03	0.09	[0.06, 0.12]	< 0.01*
Skewness GL	0.12 ± 0.04	0.16 ± 0.04	0.04	[0.005, 0.08]	0.03*
Textural features (second set)					
Energy (× 10^–3^)	5.28 ± 1.80	3.61 ± 1.92	− 1.66	[− 2.96, − 0.36]	**0.01**
Entropy	8.43 ± 0.29	8.54 ± 0.31	0.11	[− 0.12, 0.33]	0.35
Inverse difference moment	0.31 ± 0.03	0.29 ± 0.04	− 0.01	[− 0.04, 0.01]	0.33
Contrast	39.18 ± 14.64	35.49 ± 15.25	− 3.69	[− 12.03, 4.66]	0.39
Cluster shade (× 10^3^)	5.69 ± 3.71	5.33 ± 3.86	− 0.35	[− 2.47, 1.77]	0.74
Cluster prominence (× 10^3^)	1149 ± 497	873 ± 539	− 276	[− 687, 135]	0.19
Textural features (third set)					
Short run emphasis	0.88 ± 0.02	0.89 ± 0.02	0.01	[− 0.004, 0.02]	0.18
Long run emphasis	4.36 ± 0.80	3.77 ± 0.88	− 0.59	[− 1.28, 0.10]	0.09
GL non-uniformity	1727 ± 310	1701 ± 331	− 26	[− 250, 199]	0.82
Run length non-uniformity (× 10^3^)	38.59 ± 3.26	42.27 ± 3.61	3.68	[0.65, 6.71]	**0.02**
Low GL run emphasis (× 10^–3^)	2.98 ± 1.40	1.72 ± 1.52	− 1.26	[− 2.41, − 0.11]	**0.03**
High GL run emphasis	2079 ± 626	1584 ± 659	− 495	[− 898, − 92]	**0.02**
Short run low GL emphasis (× 10^–3^)	2.40 ± 1.06	1.43 ± 1.14	− 0.96	[− 1.78, − 0.14]	**0.02**
Short run high GL emphasis	1942 ± 605	1452 ± 636	− 490	[− 877, − 104]	**0.01**
Long run low GL emphasis	0.04 ± 0.04	0.02 ± 0.04	− 0.02	[− 0.05, 0.01]	0.12
Long run high GL emphasis	5176 ± 1038	4654 ± 1101	− 522	[− 1242, 198]	0.16

GL: gray level; LS mean: least squares mean; SE: standard error; CI: confidence interval.

Bold p: p < 0.05. *: q < 0.05.

### Placenta textural features and pandemic status

The comparisons of the placental textural features between pre-pandemic and pandemic epochs are shown in Supplementary Figs. 2–4 and Table 3. In the first set of textural features, mean GL was reduced while kurtosis GL and skewness GL were increased in the pandemic cohort when adjusting for GA at MRI in the GEE models (Supplementary Fig. 2). In the second set, energy was decreased in the pandemic cohort (Supplementary Fig. 3). In the third set, long run emphasis, low GL run emphasis, high GL run emphasis, short run low GL emphasis, short run high GL emphasis, and long run high GL emphasis were all decreased, while run length non-uniformity was increased in the pandemic cohort (Supplementary Fig. 4). After further adjusting for maternal distress (low vs. high distress), the pandemic status was associated with the above textural features except long run emphasis and long run high GL emphasis. (Table 3).

### Placental features and birth weight

The associations between placental features and birth weight by pandemic status are shown in Table 4. The placental volume, elongation and run length non-uniformity were positively associated with birth weight for both pre-pandemic and pandemic cohorts. In the pre-pandemic cohort, birth weight increased when cluster shade decreased while GL non-uniformity increased. In the pandemic cohort, mean GL was negatively associated with birth weight while kurtosis GL was positively associated with birth weight.

**Table 4:** The results of the generalized estimating equations for the associations between placental features and birth weight (g) by pandemic status, adjusting for GA at MRI (weeks), maternal distress (0: low distress, 1: high distress), and GA at birth (weeks).

	Pre-pandemic		Pandemic	
	$\beta$	*p*	$\beta$	*p*
Shape features				
Volume (cm^3^)	0.73	< 0.01*	2.30	< 0.01*
Thickness (mm)	4.04	0.16	5.76	0.41
Elongation (mm)	4.19	< 0.01*	7.77	< 0.01*
Textural features (first set)				
Mean GL	− 8.45	0.54	− 179.36	0.01*
Variance GL	− 18.32	0.76	− 355.36	0.38
Kurtosis GL (× 10^3^)	− 0.06	0.80	1.34	< 0.01*
Skewness GL	− 206.45	0.15	287.80	0.58
Textural features (second set)				
Energy (× 10^3^)	3.52	0.26	7.25	0.65
Entropy	− 15.52	0.47	− 45.42	0.70
Inverse difference moment	226.84	0.23	87.68	0.91
Contrast	− 0.61	0.25	1.60	0.56
Cluster shade (× 10^–3^)	− 2.57	0.01*	15.04	0.12
Cluster prominence (× 10^–6^)	− 6.46	0.52	47.57	0.28
Textural features (third set)				
Short run emphasis	− 398.01	0.21	− 365.51	0.80
Long run emphasis	10.74	0.24	8.02	0.67
GL non-uniformity	0.05	**0.04**	0.17	0.10
Run length non-uniformity (× 10^–3^)	5.12	< 0.01*	25.83	< 0.01*
Low GL run emphasis (× 10^3^)	5.63	0.09	8.00	0.20
High GL run emphasis (× 10^–3^)	− 5.84	0.67	59.67	0.37
Short run low GL emphasis (× 10^3^)	6.11	0.09	11.34	0.22
Short run high GL emphasis (× 10^–3^)	− 6.06	0.67	62.03	0.38
Long run low GL emphasis	20.57	0.86	253.72	0.20
Long run high GL emphasis (× 10^–3^)	− 1.97	0.80	32.20	0.38

GA: gestational age; GL: gray level.

Bold p: p < 0.05. *: q < 0.05.

### Mediation analyses

**Figure 3:**
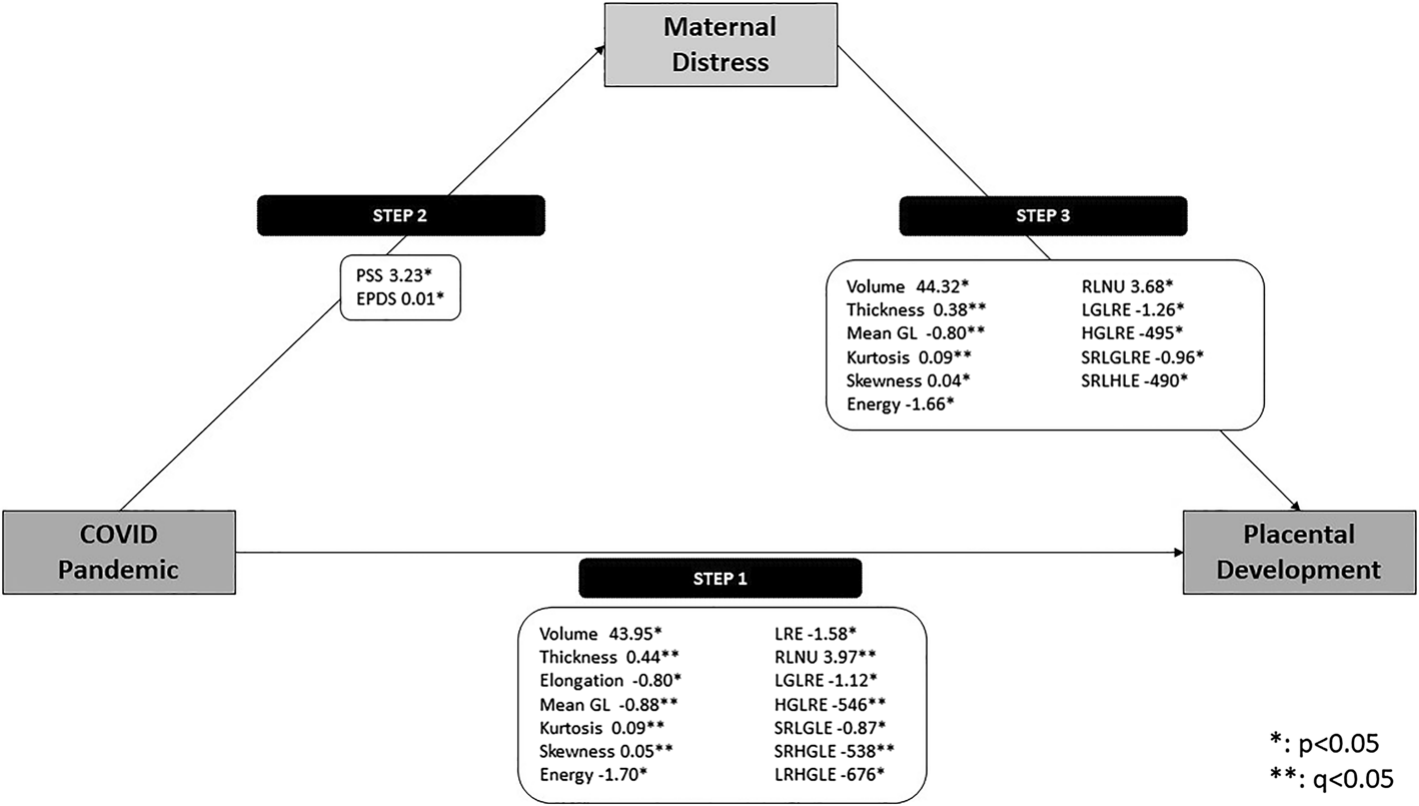
The relationship between the COVID-19 pandemic, maternal mental distress and placental development. In Step 1, COVID-19 was associated with 14 placental features, including placental morphometry (volume, thickness, elongation) and texture (mean gray level (GL), kurtosis, skewness, energy, long run emphasis (LRE), run-length non-uniformity (RLNU), Low GL run emphasis (LGLRE), high GL run emphasis (HGLRE), short run low- and high- gray level emphases (SRLGLE/SRHGLE) and long run high gray level emphasis (LRHGLE). In Step 2, COVID-19 was associated with significant increase in maternal stress and depression, compared to pre-pandemic controls. In Step 3, we re-evaluate the association between COVID-19 and placental developing while adjusting for maternal distress and found that placental volume, thickness, mean GL, kurtosis, skewness, energy, RLNU, LGLRE, HGLRE, SRGLRE and SRHLE remained significantly different between pandemic and pre-pandemic controls. Features denoted by ** (q < 0.05) highlight features that remain significant after adjusting for multiple comparisons. All steps also were adjusted for gestational age at MRI.

The mediation analyses show that in Step 1, volume, thickness, elongation, mean GL, kurtosis GL, skewness GL, energy, run length non-uniformity, high GL run emphasis, short run low GL emphasis, and short run high GL emphasis were significantly changed in the pandemic cohort after adjusting for multiple comparisons (Supplementary Table 1). In Step 2, higher PSS and EPDS values were identified in the pandemic cohort as mentioned above (Table 2). In Step 3, the significance of energy, long run emphasis, and long run high GL emphasis was altered for PSS (Supplementary Table 2), and the significance of elongation, energy, long run emphasis, and long run high GL emphasis was altered for EPDS (Supplementary Table 3). These results indicate that PSS and EPDS may mediate the association between targeted placental features and the pandemic status (Fig. 3).

### Sensitivity analyses

We conducted sensitivity analyses with additional covariates including fetal sex and maternal weight at MRI in the GEE models to evaluate the association between placental features and pandemic status. The results show that fetal sex was not a significant factor on placental shape/textural features (Supplementary Table 4). While we observed that maternal weight was significantly associated with several textural features, including mean GL, variance GL, kurtosis GL, cluster shade and cluster prominence, these findings did not change the significance of the main findings after adjusting for multiple comparisons (Supplementary Table 5).

### Time trend of placental features

We further conducted 2 GEE models (one for each cohort) to explore the association between placental features and scan date, adjusting for gestational age at MRI (weeks). The results show that within the pre-pandemic cohort, placental volume, thickness, elongation, GL non-uniformity, low GL run emphasis and short run low GL emphasis significantly increased over time. However, within the pandemic cohort, we did not identify a significant difference of placental features over time after adjusting for multiple comparisons (Supplementary Table 6). To better explore the temporal trends across both cohorts for the entire study period, the time trend was fitted using nonlinear mixed-effects estimation with the quadratic spline function. The results show that placenta thickness, kurtosis GL, and skewness GL were significantly increased while elongation, mean GL, low GL run emphasis, high GL run emphasis, short run low GL emphasis, short run high GL emphasis, long run low GL emphasis and long run high GL emphasis were significantly decreased as scan date increased, after adjusting for multiple comparisons (Supplementary Table 7), consistent with the findings in Table 3.

## Discussion

Our study aimed to assess changes in morphometric and textural features of in vivo placentas in women pregnant prior to and during the COVID-19 pandemic. We found that multiple features of placental morphometry and texture differed significantly between the two cohorts, mediated in part by the elevated maternal stress and depression identified in the pandemic cohort. Specifically, increased placental volume and thickness were observed amid the COVID-19 pandemic, and textural analyses showed asymmetry of image signal intensity (i.e., increased skewness GL and kurtosis GL) in the pandemic cohort. We also found larger inhomogeneous area (i.e., decreased long run emphasis) and higher non-uniformity (i.e., lower energy and increased run length non-uniformity) of placental images in the pandemic cohort. Furthermore, there were no significant temporal differences in placental development within the pandemic cohort for the duration of the pandemic period studied. Lastly, we report that the relationships between placental morphometry and birthweight differed in the pandemic and pre-pandemic cohorts, with stronger associations between placental volume, elongation, run length non-uniformity and birthweight during the pandemic.

### Maternal mental health and placental adaptations

Placental adaptations to perturbations in the maternal environment and mental health can trigger adverse fetal programming^48^. Studies have shown that maternal mental health disorders and prenatal stress can alter fetal development, affecting the child’s health long after the original insult such as difficult temperament, dysregulated sleep and harder-to-soothe infants, and later, lower cognitive performance and worse school achievement^49,50^. Prenatal stress has been associated with increased risk of depression, anxiety, attention-deficit/hyperactivity disorder (ADHD), conduct disorders, autism and schizophrenia^51–54^. Prior studies also have indicated the increased concerns of psychological disorders in pregnant women during the pandemic compared to non-pregnant women during the pandemic and compared to pre-pandemic pregnant women^55,56^, which may represent risks for offspring neurodevelopment including delayed cognitive, language, and motor development^57–61^. Our data showed that maternal stress and depression were elevated in the pandemic cohort, and maternal distress was a mediator of the association between pandemic status and placental morphometry and texture. In addition to detecting larger placentas in the pandemic cohort, we also found that the relationship between in vivo placental size and infant birthweight was greater when compared to pre-pandemic controls. These results build upon previous work showing that elevated maternal psychosocial stress is associated with increased placental weight at birth on evaluation of gross pathology specimen^62^. These findings demonstrated an important link between maternal mental health and the placental development, which may further influence neurodevelopmental outcomes^63–65^.

### Morphometric changes of the placenta during the pandemic

In this work, we show that the pandemic cohort had morphometric changes in the placenta that represent enlarged, globular placentas. While the mechanisms of increased placental volume and thickness are unclear, eccrinology and molecular studies suggest that epigenetic changes resulting from maternal distress may upregulate growth factors, resulting in increased placental size. Several potential mediators linking maternal stress and placental growth have been proposed. One such mechanism is that increased maternal stress could result in increased production of insulin-like growth factors, which can increase placental volume^66,67^. Secondly, cytokines are potential mediator between stress and placental volume. Psychological stress is linked to lower interleukin-10 (IL-10) during pregnancy^68^. Animal models with IL-10 deficiency showed an increase of up to 28% in placental size^69^. Epigenetic mechanisms further explain how maternal stress may affect specific placental gene expression patterns. In an animal study, an insulin-like growth factor called peroxisome proliferator-activated receptor alpha (PPARα), which binds tightly with protein 1 (IGFBP-1), hypoxia-inducible factor 3a (HIF3), and glucose transporter 4 (GLUT4), has increased expression for male offspring of pregnant mice with heightened prenatal stress^70^. Building on the existing data from previous pathology and molecular studies, in this study, we showed a similar pattern of accelerated in vivo placental volume during the COVID-19 pandemic. Further studies exploring these relationships are warranted.

An enlarged placenta has been identified as an important factor in altered maternal–fetal nutrient supply and resulting fetal programming^71^. Khalife et al. reported significant positive associations between placental size (weight, surface area, and placental-to-birth-weight ratio) and mental health problems in boys at 8 and 16 years of age. Specifically, increased placental weight was linked with overall probable psychiatric disturbance, antisocial behavior and ADHD symptoms^71^. Abnormally enlarged placentas with altered shape also have been associated with certain medical conditions, such as maternal anemia, hypertension and diabetes^72–74^.

Our data show that placental volume and elongation was positively associated with birth weight in both cohorts, and this finding is in line with several previous studies^75–81^. We also showed that the ratio of the placental volume/elongation relative to birth weight was smaller in the pandemic vs. the pre-pandemic cohort (i.e., the inverse of $\beta$ in Table 4). This reduced placental-weight-to-birth-weight (PW:BW) ratio in the setting of the COVID-19 pandemic may be associated with adverse pregnancy outcomes that result from placental insufficiency^81^. Studies have shown that a PW:BW ratio below the 10th percentile was associated with fetal distress^81^, while small-for -gestational-age infants demonstrated an elevated birth-wight-to-placental-weight ratio^82^. In addition, the highest quintile of birth weight to placental weight ratio was associated with higher uterine artery Doppler mean pulsatility index and umbilical artery Doppler pulsatility index later in gestation^23^. Major congenital anomalies have been linked to higher (> 90th) percentiles of birth-weight-to-placental-weight ratio^83^. The impact of altered placental morphometry during pandemic on infant neurodevelopmental outcomes merits further investigation.

### Textural changes of the placenta during the pandemic

We found that several textural features were significantly different in the pandemic cohort, namely features associated with asymmetry of gray scale and image heterogeneity. Specifically, we noted an increase in heterogeneity and asymmetry of image intensity of placentas in the pandemic cohort.

Textural analyses of sonographic and MR images of the placenta have shown increased heterogeneity with advancing GA, a reflection of the increasing complexity of placental microstructure during gestation^24,84,85^. Regional changes in placental texture have been observed in placenta accrete^86–88^, while global changes in placental texture have been observed in fetal growth restriction (FGR)^24^. In this work, we found a decrease in mean GL and SRLGL along with increased kurtosis and skewness in the pandemic cohort, the converse of findings previously reported in FGR^24^. We also report an increase in RLNU during the pandemic, similar to changes reported in FGR, along with previously undescribed decreases in LGLRE, HGLRE and SRHGLE. The precise mechanisms of the microstructural changes of placentas observed from textural analyses in the pandemic cohort remain largely unclear and may in fact be multi-factorial. Previous studies have collectively pointed toward maternal psychosocial stress pathways that may alter the epigenetic signature in placentas^89–94^. First, prenatal maternal stress is linked with higher levels of maternal cortisol^89^, and the increase in maternal cortisol alters uteroplacental metabolites such as serotonin^90,95^, which is associated with beta cell heterogeneity in tryptophan hydroxylase protein induction during pregnancy^91^. Second, differential expression of placental glucocorticoid receptors has been reported in the presence of elevated maternal stress^92,93^, and a study has shown that glucocorticoid treatment could lead to increased glucocorticoid receptor mRNA variants detected in the human placenta, where GR-α and GR-1C mRNA having the highest expression^96^. Third, placental DNA methylation changes are associated with increased exposure to maternal stress^94,97,98^. These DNA methylation changes could regulate placental-specific gene expression, including monoallelic expression and X-chromosome inactivation in the placenta, leading to the changes of placental context^94,97,98^. These alterations in placental metabolism and gene expression associated with maternal distress may result in microstructural changes that can be detected by texture analyses and deserve further clinical validation particularly for the pandemic population.

Our data show that altered GL measures, namely decreased mean GL and increased kurtosis GL, of the placenta were associated with increased birth weight in the pandemic cohort, which may reflect altered placental microstructure; while the etiology of these changes is not fully elucidated, these may be related to pandemic related physiologic, psychologic and environmental stressors. The placenta can undergo major structural and functional adaptations to shield the fetus from environmental stressors^99–101^. Genome-wide placental transcriptome studies have correlated gene modules involved in immune response, myeloid cell differentiation, and placental tissue development with newborn birth weight^102^. The placenta is genetically identical with the fetus^103,104^, and epidemiological studies suggest that genetic factors account for 30–80% of birth weight variance^105–107^. Interestingly, placental DNA methylation may be influenced by maternal insulin levels during pregnancy^108^, and increased placental DNA methylation is associated with large-for-gestational-age infants^109^, and methylation alternations may reflect changes to placental texture^110^. Nevertheless, the mechanisms underlying these observations need further interrogation.

## Strengths and limitations

Our strengths include a novel, non-invasive approach to analyze in vivo placental morphometry and texture to detect early alterations in pregnant women affected by the COVID-19 pandemic mediated, at least in part, by maternal mental distress. We utilized the 3-D reconstructed models and computational analyses of texture based on high-resolution MR images to detect abnormalities, representing gross and microstructural changes in placental development during the pandemic^111,112^. The benefits of MRI lead to finer 3-D placental models and better estimation of the morphometric and textural features of human placentas presented in our study.

Although our study had several strengths, the study limitations need to be outlined. First, the segmentation of the placentas from MR images was performed manually. Due to the variability of the placental shape, orientation and appearance, the fully automatic segmentation of the placenta remains a challenge^46,113^. Thus, manual segmentation is currently the best method used in the literature and has served as the ground truth when developing segmentation algorithms for the human placenta^20,114,115^. Second, this work was intended to explore the impact of the pandemic on placental development and included women with no known COVID-19 exposures, based on serial questionnaires. However, subclinical or unknown exposures to the COVID-19 virus cannot be fully excluded. Similarly, we investigated the role of pandemic-related maternal mental distress associated with COVID-19 on placental development; however, there may be additional stressors and lifestyle changes related to the pandemic, including environmental stressors, and changes in physical activity, that also may have contributed to disrupted placental development. Furthermore, pregnant women recruited into this study were from the Washington, DC metropolitan area and the associations observed in this study should be explored in other geographic regions before assessing the generalizability of these findings. Third, the clinical implications of altered placental morphometry and texture on child neurodevelopmental for pregnancy during pandemic remain uncertain; ongoing studies of pregnancy outcome are currently underway. These include correlating of in vivo findings with ex vivo placental pathology, as well as long-term neurodevelopmental outcomes of children born during the pandemic. Lastly, application of these analyses and results should be linked to clinical evaluation and interpretation, in order to develop novel prediction models of in vivo placental structure relative to clinically significant outcomes^116–118^.

## Conclusion

Our study is the first to describe MRI-based morphometric and textural changes of in vivo placenta in women pregnant during the COVID-19 pandemic. Our approach provides a semiautomated method of standardizing placental MR evaluation in women during and prior to the pandemic. With these sophisticated techniques, we can detect in vivo changes of the placenta not only on gross-level evaluation (i.e., morphometry) but also microscopic-level assessment (i.e., texture). Moreover, we demonstrate changes in gross and microscopic placental structure in women with increased levels of prenatal stress, a potentially modifiable risk factor that, if recognized early, may allow for timely interventions to improve placental health and pregnancy outcomes. Future studies relating in vivo measures of placental development with clinical evaluations and outcomes may lead to the development of clinically relevant prediction models of placental health^116–118^. The further evaluation of these findings with placental pathology, pregnancy outcomes and long-term neurodevelopmental health is currently under investigation.

## Supplementary Information

Supplementary Figures.

Supplementary Tables.

## Data Availability

The datasets used and/or analyzed during the current study available from the corresponding author on reasonable request.
