# Supplementary material for: Influence of maternal psychological distress during COVID-19 pandemic on placental morphometry and texture
Source: Sci Rep. 2023 May 10;13:7374. doi: 10.1038/s41598-023-33343-4 (PMC10172401; doi:10.1038/s41598-023-33343-4)
Supplement: Supplementary file 1 — Supplementary Figures. [file 41598_2023_33343_MOESM1_ESM.docx]

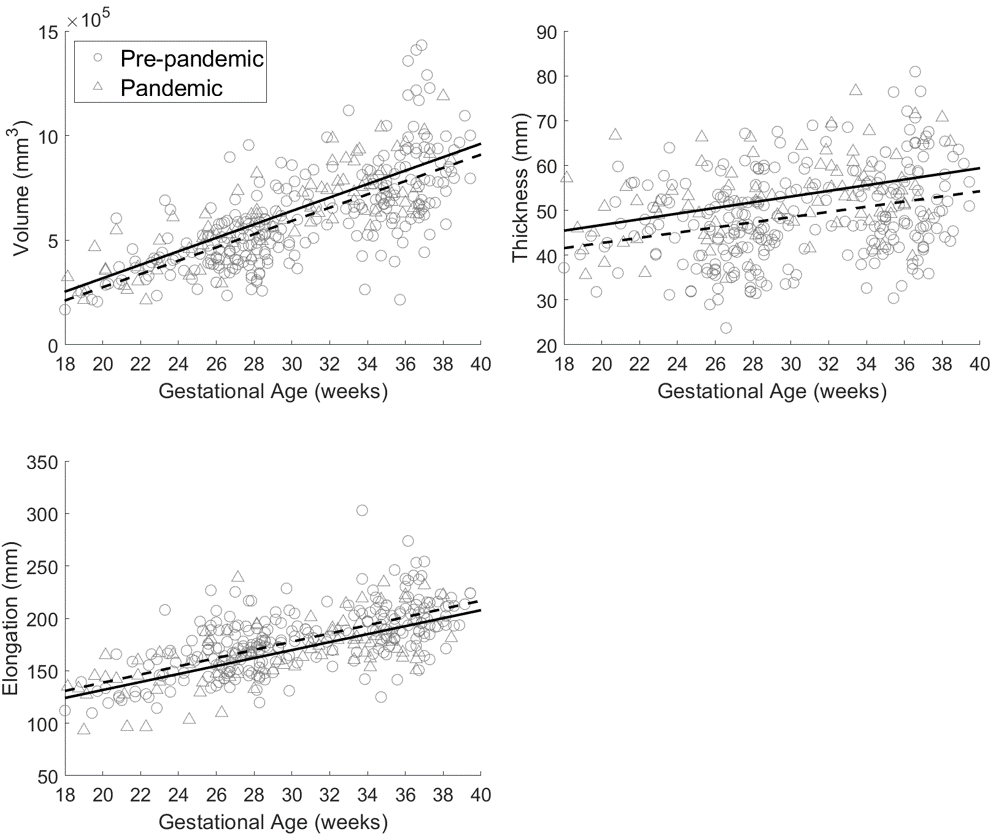


**Supplementary Figure 1.** Scatter plots of placental shape features vs. gestational age. (a) Volume; (b) Thickness; (c) Elongation. Dashed line: Linear fit of pre-pandemic data. Solid line: Linear fit of pandemic data. Bold p: p<0.05. *: q<0.05.

(a)

(b)

(c)

$\mathbf{p}$**=0.02***

$\mathbf{p}$**<0.01***

$\mathbf{p}$**=0.01***


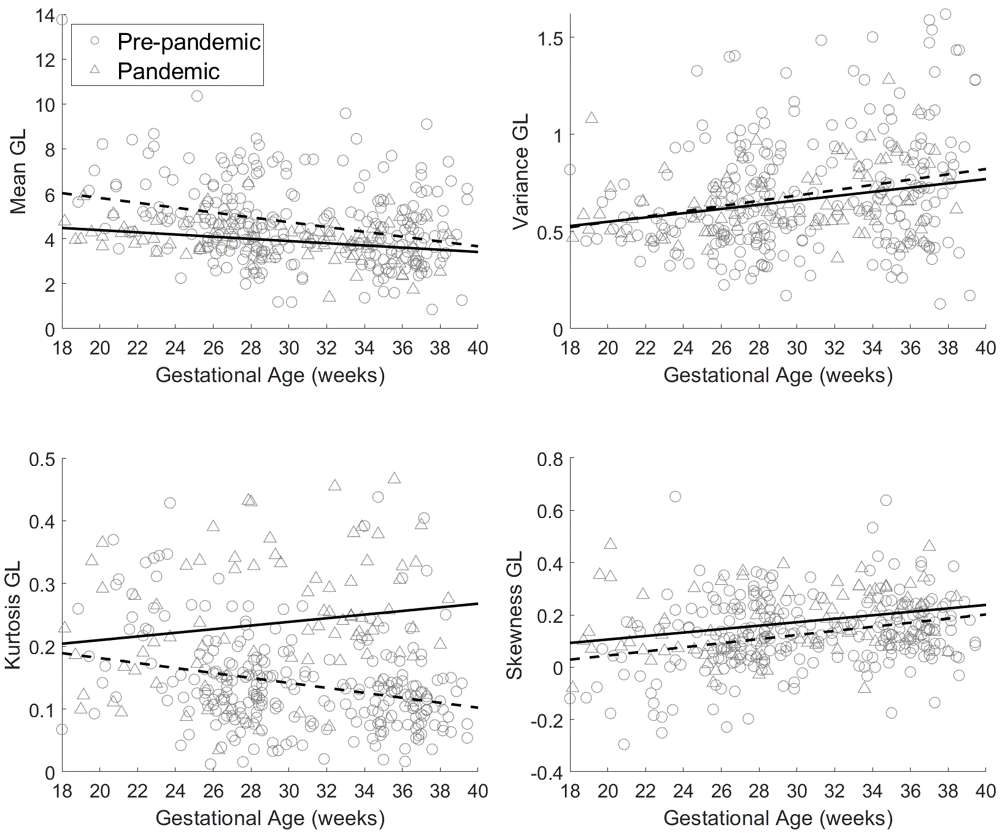


**Supplementary Figure 2.** Scatter plots of first set of placental textural features vs. gestational age. (a) Mean GL; (b) Variance GL; (c) Kurtosis GL; (d) Skewness GL. Dashed line: Linear fit of pre-pandemic data. Solid line: Linear fit of pandemic data. Bold p: p<0.05. *: q<0.05.

(a)

(b)

(c)

$\mathbf{p}$**<0.01***

$\mathbf{p}$**=0.51**

$\mathbf{p}$**<0.01***

(d)

$\mathbf{p}$**<0.01***


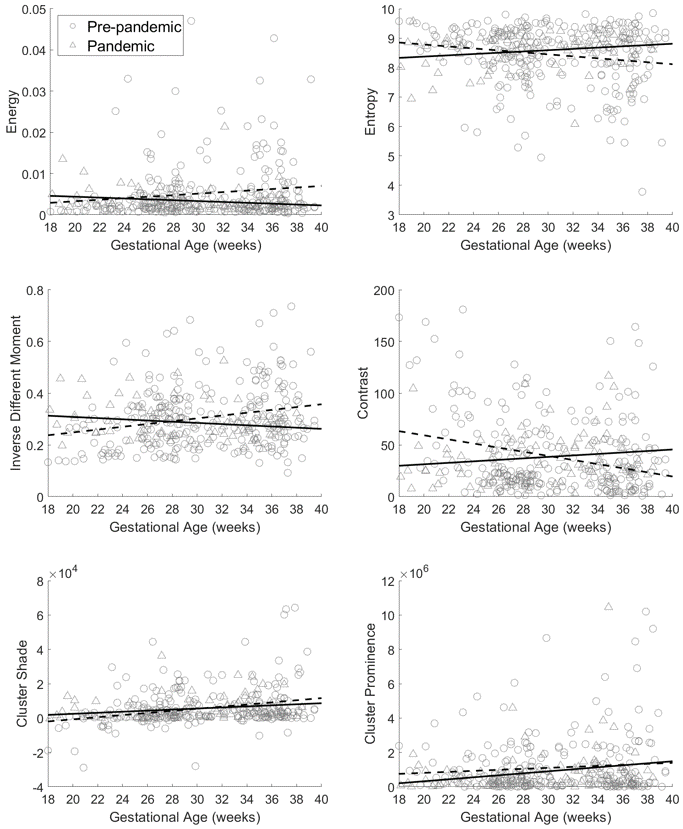


**Supplementary Figure 3.** Scatter plots of second set of placental textural features vs. gestational age. (a) Energy; (b) Entropy; (c) Inverse difference moment; (d) Contrast; (e) Cluster shade; (f) Cluster prominence. Dashed line: Linear fit of pre-pandemic data. Solid line: Linear fit of pandemic data. Bold p: p<0.05. *: q<0.05.

(a)

(b)

(c)

(d)

(e)

(f)

$\mathbf{p}$**<0.01***

$p$=0.24

$p$=0.69

$p$=0.23

$p$=0.54

$p$=0.33


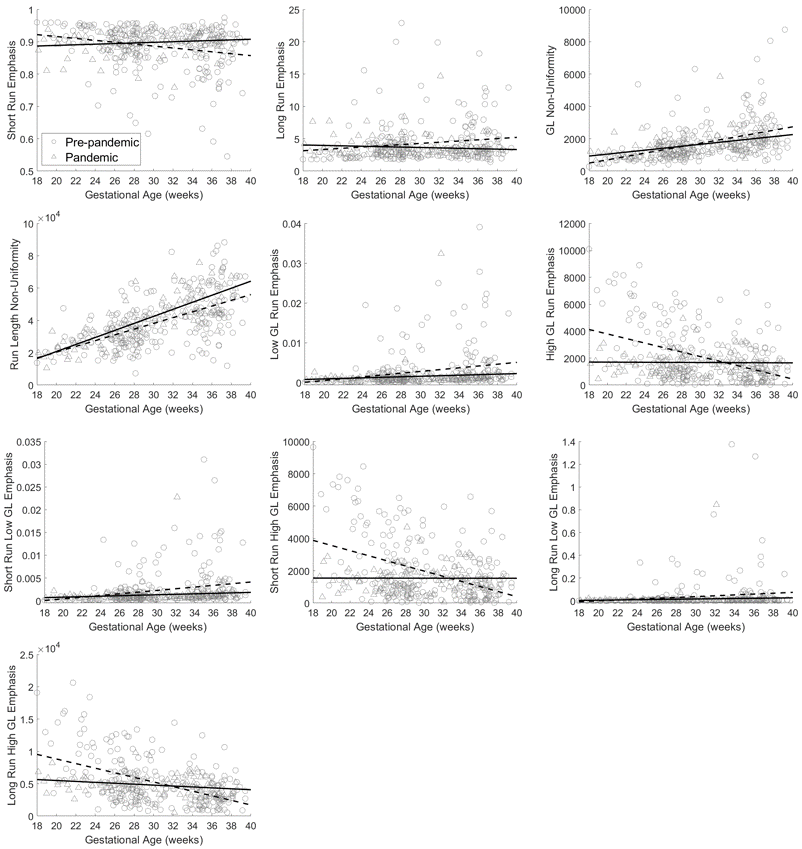


**Supplementary Figure 4.** Scatter plots of third set of placental textural features vs. gestational age. (a) Short run emphasis; (b) Long run emphasis; (c) Gray level non-uniformity; (d) Run length non-Uniformity; (e) Low gray level run emphasis; (f) High gray level run emphasis; (g) Short run low gray level emphasis; (h) Short run high gray level emphasis; (i) Long run low gray level emphasis; (j) Long run high gray level emphasis. GL: Gray level. Dashed line: Linear fit of pre-pandemic data. Solid line: Linear fit of pandemic data. Bold p: p<0.05. *: q<0.05.

(a)

(b)

(c)

(d)

(e)

(f)

(g)

(h)

(i)

(j)

$p$=0.11

$\mathbf{p}$**<0.01***

$\mathbf{p}$**=0.02***

$\mathbf{p}$**=0.04**

$\mathbf{p}$**=0.047**

$\mathbf{p}$**=0.03**

$\mathbf{p}$**<0.01***

$p$=0.63

$\mathbf{p}$**<0.01***

$p$=0.13

**Supplementary Figure 5.** The histogram of number of subjects recruited in each month: (a) Pre-pandemic cohort; (b) Pandemic cohort.


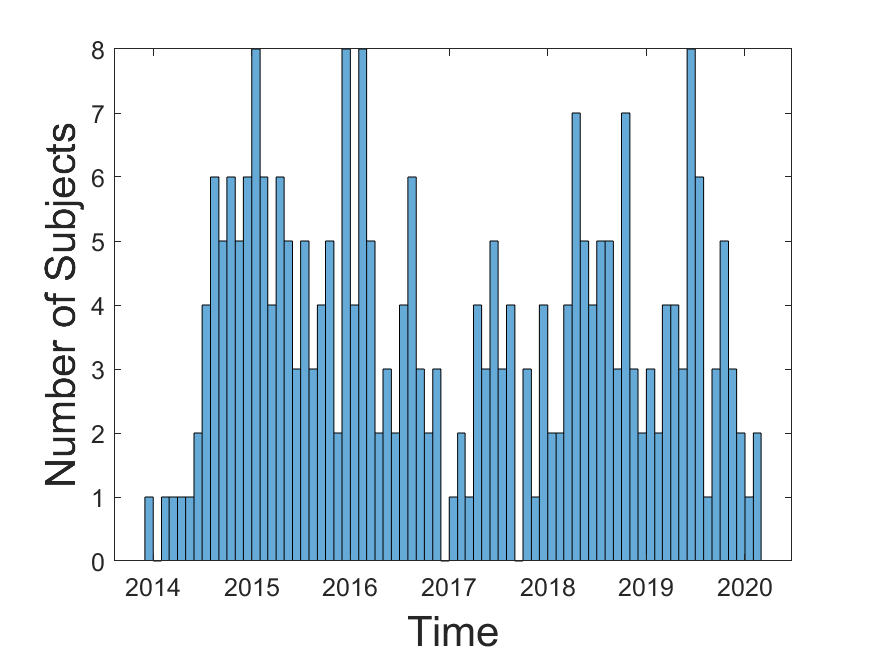


(a)


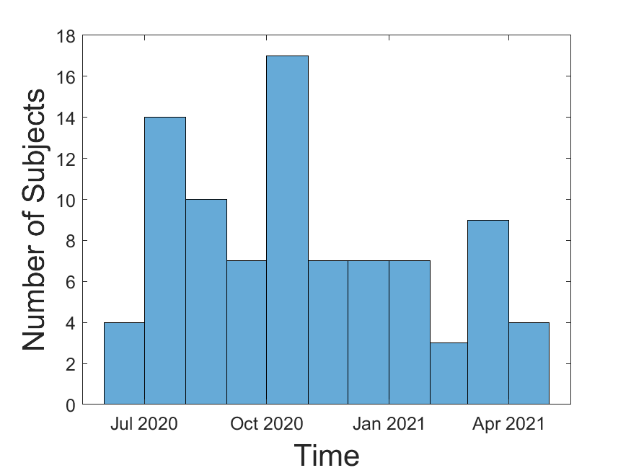


(b)
