# Supplementary material for: Influence of maternal psychological distress during COVID-19 pandemic on placental morphometry and texture
Source: Sci Rep. 2023 May 10;13:7374. doi: 10.1038/s41598-023-33343-4 (PMC10172401; doi:10.1038/s41598-023-33343-4)
Supplement: Supplementary file 2 — Supplementary Tables. [file 41598_2023_33343_MOESM2_ESM.docx]

**Supplementary Table 1**. The results of the generalized estimating equations for the associations between placental shape/textural features and pandemic status (0: pre-pandemic; 1: pandemic), adjusting for gestational age at MRI (weeks).

|  | Pre-pandemic  (LS Mean±SE) | Pandemic  (LS Mean±SE) | $\beta$ | 95% CI | p |
| --- | --- | --- | --- | --- | --- |
| Shape Features | | | | | |
| Volume (cm^3^) | 594.0±39.6 | 637.9±43.7 | 43.95 | [8.00, 79.90] | **0.02*** |
| Thickness (cm) | 4.9±0.2 | 5.3±0.2 | 0.44 | [0.20, 0.67] | **<0.01*** |
| Elongation (cm) | 17.8±0.6 | 17.0±0.7 | -0.80 | [-1.43, -0.17] | **0.01*** |
| Textural Features (First Set) | | | | | |
| Mean GL | 4.74±0.49 | 3.86±0.51 | -0.88 | [-1.20, -0.56] | **<0.01*** |
| Variance GL | 0.68±0.08 | 0.67±0.08 | -0.02 | [-0.07, 0.04] | 0.51 |
| Kurtosis GL | 0.14±0.02 | 0.23±0.03 | 0.09 | [0.07, 0.12] | **<0.01*** |
| Skewness GL | 0.12±0.04 | 0.17±0.04 | 0.05 | [0.02, 0.08] | **<0.01*** |
| Textural Features (Second Set) | | | | | |
| Energy (×10^-3^) | 5.16±1.70 | 3.46±1.80 | -1.70 | [-2.89, -0.51] | **<0.01*** |
| Entropy | 8.45±0.26 | 8.57±0.28 | 0.12 | [-0.08, 0.32] | 0.23 |
| Inverse difference moment | 0.30±0.03 | 0.29±0.03 | -0.01 | [-0.04, 0.01] | 0.24 |
| Contrast | 39.62±12.76 | 37.19±13.37 | -2.43 | [-10.26, 5.40] | 0.54 |
| Cluster shade (×10^3^) | 5.42±3.22 | 5.82±3.37 | 0.39 | [-1.55, 2.33] | 0.69 |
| Cluster prominence (×10^3^) | 1102±435 | 918±474 | -184 | [-556, 188] | 0.33 |
| Textural Features (Third Set) | | | | | |
| Short run emphasis | 0.89±0.02 | 0.90±0.02 | 0.01 | [-0.002, 0.02] | 0.11 |
| Long run emphasis | 4.29±0.71 | 3.71±0.77 | -0.58 | [-1.15, -0.01] | **0.047** |
| GL non-uniformity | 1720±304 | 1669±322 | -51 | [-257, 155] | 0.63 |
| Run length non-uniformity (×10^3^) | 38.25±2.98 | 42.22±3.28 | 3.97 | [1.27, 6.66] | **<0.01*** |
| Low GL run emphasis (×10^-3^) | 2.83±1.28 | 1.71±1.38 | -1.12 | [-2.13, -0.10] | **0.03** |
| High GL run emphasis | 2136±553 | 1590±582 | -546 | [-903, -189] | **<0.01*** |
| Short run low GL emphasis (×10^-3^) | 2.28±0.97 | 1.41±1.04 | -0.87 | [-1.59, -0.15] | **0.02*** |
| Short run high GL emphasis | 1996±534 | 1458±562 | -538 | [-881, -195] | **<0.01*** |
| Long run low GL emphasis | 0.04±0.03 | 0.02±0.03 | -0.02 | [-0.04, 0.01] | 0.13 |
| Long run high GL emphasis | 5298±920 | 4622±975 | -676 | [-1308, -43] | **0.04** |

GL: Gray level. LS Mean: Least squares mean. SE: Standard error. CI: Confidence interval. Bold p: p<0.05. *: q<0.05.

**Supplementary Table 2.** The results of the generalized estimating equations for the associations between placental shape/textural features and pandemic status (0: pre-pandemic; 1: pandemic), adjusting for gestational age at MRI (weeks) and PSS.

|  | Pandemic status | | PSS | |
| --- | --- | --- | --- | --- |
|  | $\beta$ | p | $\beta$ | p |
| Shape Features | | | | |
| Volume (cm^3^) | 42.50 | **0.04*** | -0.85 | 0.60 |
| Thickness (cm) | 0.42 | **<0.01*** | 0.01 | 0.41 |
| Elongation (cm) | -0.79 | **0.03*** | 0.02 | 0.48 |
| Textural Features (First Set) | | | | |
| Mean GL | -0.89 | **<0.01*** | 0.01 | 0.59 |
| Variance GL | -0.05 | 0.12 | 0.004 | 0.12 |
| Kurtosis GL | 0.09 | **<0.01*** | -0.002 | **0.02** |
| Skewness GL | 0.04 | **0.01*** | -0.0003 | 0.80 |
| Textural Features (Second Set) | | | | |
| Energy (×10^-3^) | -1.66 | **0.02** | 0.0002 | 0.997 |
| Entropy | 0.08 | 0.46 | 0.01 | 0.57 |
| Inverse difference moment | -0.01 | 0.34 | 0.0004 | 0.74 |
| Contrast | -3.32 | 0.43 | -0.21 | 0.55 |
| Cluster shade (×10^3^) | -0.48 | 0.64 | 0.09 | 0.41 |
| Cluster prominence (×10^3^) | -296 | 0.10 | 6.73 | 0.71 |
| Textural Features (Third Set) | | | | |
| Short run emphasis | 0.01 | 0.18 | -0.0002 | 0.79 |
| Long run emphasis | -0.65 | 0.07 | 0.04 | 0.23 |
| GL non-uniformity | 8.51 | 0.94 | -15.9 | 0.14 |
| Run length non-uniformity (×10^3^) | 3.58 | **0.02*** | -0.05 | 0.75 |
| Low GL run emphasis (×10^-3^) | -1.34 | **0.02*** | 0.07 | 0.15 |
| High GL run emphasis | -522 | **0.01*** | -8.27 | 0.60 |
| Short run low GL emphasis (×10^-3^) | -1.00 | **0.01*** | 0.04 | 0.28 |
| Short run high GL emphasis | -516 | **0.01*** | -7.76 | 0.61 |
| Long run low GL emphasis | -0.03 | 0.07 | 0.002 | 0.052 |
| Long run high GL emphasis | -594 | 0.10 | -20.1 | 0.48 |

GL: Gray level. Bold p: p<0.05. *: q<0.05.

**Supplementary Table 3.** The results of the generalized estimating equations for the associations between placental shape/textural features and pandemic status (0: pre-pandemic; 1: pandemic), adjusting for gestational age at MRI (weeks) and EPDS.

|  | Pandemic status | | EPDS | |
| --- | --- | --- | --- | --- |
|  | $\beta$ | p | $\beta$ | p |
| Shape Features | | | | |
| Volume (cm^3^) | 43.24 | **0.03*** | 1.48 | 0.53 |
| Thickness (cm) | 0.39 | **<0.01*** | 0.01 | 0.30 |
| Elongation (cm) | -0.67 | 0.052 | 0.07 | 0.07 |
| Textural Features (First Set) | | | | |
| Mean GL | -0.86 | **<0.01*** | 0.02 | 0.52 |
| Variance GL | -0.04 | 0.15 | 0.01 | **0.02*** |
| Kurtosis GL | 0.09 | **<0.01*** | -0.003 | **0.01*** |
| Skewness GL | 0.05 | **0.02*** | 0.0001 | 0.97 |
| Textural Features (Second Set) | | | | |
| Energy (×10^-3^) | -1.77 | **0.01** | 0.02 | 0.83 |
| Entropy | 0.10 | 0.36 | 0.01 | 0.49 |
| Inverse difference moment | -0.01 | 0.32 | 0.0006 | 0.74 |
| Contrast | -3.94 | 0.36 | -0.22 | 0.69 |
| Cluster shade (×10^3^) | -0.17 | 0.87 | 0.09 | 0.53 |
| Cluster prominence (×10^3^) | -257 | 0.20 | -1.18 | 0.96 |
| Textural Features (Third Set) | | | | |
| Short run emphasis | 0.01 | 0.16 | -0.0002 | 0.85 |
| Long run emphasis | -0.65 | 0.06 | 0.06 | 0.20 |
| GL non-uniformity | -26.27 | 0.81 | -14.9 | 0.38 |
| Run length non-uniformity (×10^3^) | 3.80 | **0.01*** | 0.04 | 0.83 |
| Low GL run emphasis (×10^-3^) | -1.37 | **0.01*** | 0.14 | 0.13 |
| High GL run emphasis | -548 | **0.01*** | -5.31 | 0.85 |
| Short run low GL emphasis (×10^-3^) | -1.00 | **0.01*** | 0.06 | 0.40 |
| Short run high GL emphasis | -541 | **0.01*** | -4.96 | 0.85 |
| Long run low GL emphasis | -0.03 | 0.07 | 0.005 | 0.07 |
| Long run high GL emphasis | -626 | 0.09 | -14.5 | 0.78 |

GL: Gray level. Bold p: p<0.05. *: q<0.05.

**Supplementary Table 4.** The results of the generalized estimating equations for the associations between placental shape/textural features and pandemic status (0: pre-pandemic; 1: pandemic), adjusting for gestational age at MRI (weeks) and fetal sex (0: female; 1: male).

|  | Pandemic status | | Fetal sex | |
| --- | --- | --- | --- | --- |
|  | $\beta$ | p | $\beta$ | p |
| Shape Features | | | | |
| Volume (cm^3^) | 47.7 | **0.01*** | 22.3 | 0.25 |
| Thickness (cm) | 0.43 | **<0.01*** | 0.03 | 0.81 |
| Elongation (cm) | -0.84 | **0.01*** | 0.68 | **0.03** |
| Textural Features (First Set) | | | | |
| Mean GL | -0.88 | **<0.01*** | -0.18 | 0.36 |
| Variance GL | -0.02 | 0.42 | -0.04 | 0.21 |
| Kurtosis GL | 0.10 | **<0.01*** | 0.002 | 0.86 |
| Skewness GL | 0.05 | **0.01*** | 0.01 | 0.50 |
| Textural Features (Second Set) | | | | |
| Energy (×10^-3^) | -2.02 | **0.01*** | 0.81 | 0.42 |
| Entropy | 0.13 | 0.24 | -0.17 | 0.16 |
| Inverse difference moment | -0.01 | 0.30 | 0.02 | 0.09 |
| Contrast | -4.14 | 0.34 | -6.06 | 0.20 |
| Cluster shade (×10^3^) | 0.20 | 0.85 | -0.72 | 0.54 |
| Cluster prominence (×10^3^) | -221 | 0.27 | -312 | 0.13 |
| Textural Features (Third Set) | | | | |
| Short run emphasis | 0.01 | 0.15 | -0.01 | 0.10 |
| Long run emphasis | -1.04 | **0.01*** | 0.72 | 0.22 |
| GL non-uniformity | -42.4 | 0.70 | 164 | 0.17 |
| Run length non-uniformity (×10^3^) | 4.00 | **<0.01*** | -0.43 | 0.77 |
| Low GL run emphasis (×10^-3^) | -1.89 | **0.01*** | 0.20 | 0.85 |
| High GL run emphasis | -505 | **0.01*** | -339 | 0.11 |
| Short run low GL emphasis (×10^-3^) | -1.20 | **0.01*** | 0.18 | 0.77 |
| Short run high GL emphasis | -500 | **0.01*** | -327 | 0.11 |
| Long run low GL emphasis | -0.11 | **0.02*** | 0.01 | 0.93 |
| Long run high GL emphasis | -566 | 0.09 | -496 | 0.19 |

GL: Gray level. Bold p: p<0.05. *: q<0.05.

**Supplementary Table 5.** The results of the generalized estimating equations for the associations between placental shape/textural features and pandemic status (0: pre-pandemic; 1: pandemic), adjusting for gestational age at MRI (weeks) and maternal weight at MRI (kg).

|  | Pandemic status | | Maternal weight | |
| --- | --- | --- | --- | --- |
|  | $\beta$ | p | $\beta$ | p |
| Shape Features | | | | |
| Volume (cm^3^) | 46.4 | **0.01*** | 1.12 | 0.09 |
| Thickness (cm) | 0.41 | **<0.01*** | 0.004 | 0.29 |
| Elongation (cm) | -0.74 | **0.02*** | 0.01 | 0.48 |
| Textural Features (First Set) | | | | |
| Mean GL | -0.85 | **<0.01*** | -0.04 | **<0.01*** |
| Variance GL | -0.01 | 0.62 | -0.005 | **<0.01*** |
| Kurtosis GL | 0.09 | **<0.01*** | 0.001 | **0.01*** |
| Skewness GL | 0.05 | **<0.01*** | -0.0005 | 0.38 |
| Textural Features (Second Set) | | | | |
| Energy (×10^-3^) | -1.79 | **<0.01*** | 0.05 | 0.09 |
| Entropy | 0.14 | 0.16 | -0.01 | 0.10 |
| Inverse difference moment | -0.02 | 0.19 | 0.001 | 0.26 |
| Contrast | -2.12 | 0.60 | -0.11 | 0.44 |
| Cluster shade (×10^3^) | 0.53 | 0.59 | -0.14 | **<0.01*** |
| Cluster prominence (×10^3^) | -163 | 0.39 | -18.27 | **0.01*** |
| Textural Features (Third Set) | | | | |
| Short run emphasis | 0.01 | 0.08 | -0.0003 | 0.22 |
| Long run emphasis | -0.61 | **0.04** | 0.02 | 0.09 |
| GL non-uniformity | -64.1 | 0.53 | 10.36 | **0.01** |
| Run length non-uniformity (×10^3^) | 4.27 | **<0.01*** | -0.02 | 0.78 |
| Low GL run emphasis (×10^-3^) | -1.16 | **0.02*** | 0.04 | 0.12 |
| High GL run emphasis | -536 | **<0.01*** | -5.06 | 0.51 |
| Short run low GL emphasis (×10^-3^) | -0.91 | **0.01*** | 0.01 | 0.40 |
| Short run high GL emphasis | -528 | **<0.01*** | -4.83 | 0.51 |
| Long run low GL emphasis | -0.02 | 0.12 | 0.001 | 0.11 |
| Long run high GL emphasis | -650 | **0.04** | -8.88 | 0.51 |

GL: Gray level. Bold p: p<0.05. *: q<0.05.

**Supplementary Table 6.** The results of the generalized estimating equations for the associations between placental shape/textural features and scan date, adjusting for gestational age at MRI (weeks) for the pre-pandemic cohort and the pandemic cohort.

|  | Pre-pandemic | | Pandemic | |
| --- | --- | --- | --- | --- |
|  | $\beta_{ScanDate}$ | $p_{ScanDate}$ | $\beta_{ScanDate}$ | $p_{ScanDate}$ |
| Shape Features | | | | |
| Volume (cm^3^) | 0.06 | **<0.01*** | 0.24 | 0.16 |
| Thickness (cm) | 0.0003 | **0.02*** | 0.002 | **0.04** |
| Elongation (cm) | 0.001 | **0.01*** | -0.002 | 0.57 |
| Textural Features (First Set) | | | | |
| Mean GL | 0.0001 | 0.73 | -0.001 | 0.48 |
| Variance GL | 0.00003 | 0.37 | 0.0001 | 0.69 |
| Kurtosis GL | 0.00001 | 0.34 | 0.00002 | 0.88 |
| Skewness GL | -0.00002 | 0.19 | 0.0003 | 0.11 |
| Textural Features (Second Set) | | | | |
| Energy (×10^-3^) | 0.003 | **0.01** | 0.002 | 0.72 |
| Entropy | -0.0002 | 0.10 | -0.0002 | 0.87 |
| Inverse difference moment | 0.00002 | 0.14 | -0.00001 | 0.96 |
| Contrast | 0.01 | 0.20 | 0.02 | 0.59 |
| Cluster shade (×10^3^) | -0.002 | 0.07 | 0.0001 | 0.99 |
| Cluster prominence (×10^3^) | -0.32 | **0.04** | -1.62 | 0.45 |
| Textural Features (Third Set) | | | | |
| Short run emphasis | -0.00002 | **0.03** | -0.000002 | 0.98 |
| Long run emphasis | 0.001 | **0.04** | 0.001 | 0.75 |
| GL non-uniformity | 0.34 | **0.01*** | 1.20 | 0.37 |
| Run length non-uniformity (×10^3^) | 0.001 | 0.45 | 0.01 | 0.44 |
| Low GL run emphasis (×10^-3^) | 0.002 | **<0.01*** | 0.01 | 0.25 |
| High GL run emphasis | 0.17 | 0.48 | -1.04 | 0.38 |
| Short run low GL emphasis (×10^-3^) | 0.002 | **<0.01*** | 0.01 | 0.24 |
| Short run high GL emphasis | 0.17 | 0.46 | -0.95 | 0.40 |
| Long run low GL emphasis | 0.000 | **0.02** | 0.0003 | 0.30 |
| Long run high GL emphasis | 0.05 | 0.90 | -3.63 | 0.09 |

GL: Gray level. Bold p: p<0.05. *: q<0.05.

**Supplementary Table 7.** The results of the nonlinear mixed-effects estimation with the quadratic spline function for the associations between placental shape/textural features and scan date (I.e., placental features=β_1_∙ScanDate^2^+ β_2_∙ScanDate + β_3_) for the entire period.

|  | $\beta_{1}$ (×10^-6^) | SE of $\beta_{1}$ (×10^-6^) | $p_{1}$ |
| --- | --- | --- | --- |
| Shape Features | | | |
| Volume (cm^3^) | -3.25 | 24.5 | 0.89 |
| Thickness (cm) | 0.27 | 0.12 | **0.02*** |
| Elongation (cm) | -1.13 | 0.33 | **<0.01*** |
| Textural Features (First Set) | | | |
| Mean GL | -0.80 | 0.19 | **<0.01*** |
| Variance GL | -0.03 | 0.03 | 0.29 |
| Kurtosis GL | 0.04 | 0.01 | **<0.01*** |
| Skewness GL | 0.06 | 0.02 | **<0.01*** |
| Textural Features (Second Set) | | | |
| Energy (×10^-3^) | -1.66 | 0.76 | **0.03** |
| Entropy | 0.10 | 0.11 | 0.36 |
| Inverse difference moment | -0.01 | 0.01 | 0.41 |
| Contrast | -7.63 | 4.36 | 0.08 |
| Cluster shade (×10^3^) | 2.92 | 1.11 | **0.01** |
| Cluster prominence (×10^3^) | 114 | 177 | 0.52 |
| Textural Features (Third Set) | | | |
| Short run emphasis | 0.01 | 0.01 | 0.12 |
| Long run emphasis | -0.71 | 0.35 | **0.04** |
| GL non-uniformity | -135 | 120 | 0.26 |
| Run length non-uniformity (×10^3^) | 1.74 | 1.67 | 0.30 |
| Low GL run emphasis (×10^-3^) | -1.31 | 0.55 | **0.02*** |
| High GL run emphasis | -626 | 235 | **0.01*** |
| Short run low GL emphasis (×10^-3^) | -1.03 | 0.40 | **0.01*** |
| Short run high GL emphasis | -610 | 226 | **0.01*** |
| Long run low GL emphasis | -0.03 | 0.01 | **0.01*** |
| Long run high GL emphasis | -967 | 429 | **0.02*** |

GL: Gray level. SE: Standard error. Bold p: p<0.05. *: q<0.05.
